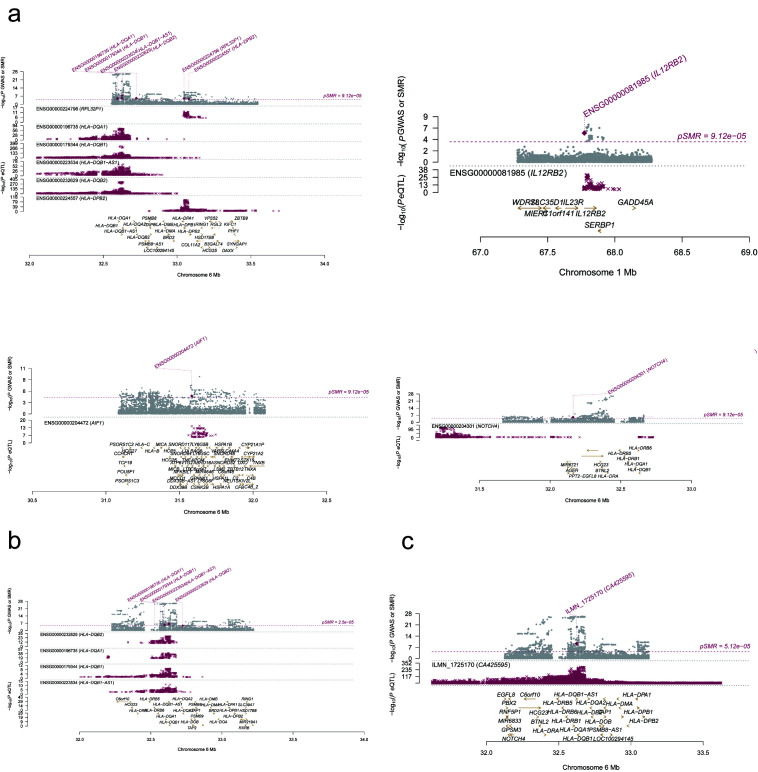# Genetic mechanisms of primary biliary cholangitis and its association with immune cells using mendelian randomization and biological annotation

**DOI:** 10.1097/MD.0000000000049146

**Published:** 2026-05-29

**Authors:** Yukai Lin, Xiaolong Xu, Hao Chen, Haifei Tang, Yanming Hua, Jianfeng Mei

**Affiliations:** a Department of Hepatological Surgery, The Second Affiliated Hospital of Zhejiang University School of Medicine, Lanxi Branch (Lanxi People’s Hospital), Jinhua, Zhejiang, China; b Medical Clinical Laboratory Center, The Second Affiliated Hospital of Zhejiang University School of Medicine, Lanxi Branch (Lanxi People’s Hospital), Jinhua, Zhejiang, China.

**Keywords:** drug targets, immune cells, Mendelian randomization, primary biliary cholangitis

## Abstract

The incidence of primary biliary cholangitis (PBC) is on the rise, attributed to autoimmune-mediated bile duct damage in genetically susceptible individuals triggered by environmental factors. The close association between immune cell infiltration around the intrahepatic bile ducts and bile duct damage, and the causal and potential relationships of immune cells in the pathogenesis of PBC, remain unclear. Utilizing summary statistics from published genome-wide association studies, we evaluated the association between 731 immune cell traits and PBC through 2-sample Mendelian randomization (MR) analysis. Through pathway enrichment and functional analysis of associated genes, we aim to predict potential mechanisms. Genomic risk loci for PBC were identified, and summary data-based Mendelian randomization (SMR) analysis was conducted to pinpoint potential genes and therapeutic targets. Our analysis revealed a significant association between the elevated proportion of IgD- CD27- %lymphocytes and reduced risk of PBC after FDR adjustment in the B-cell panels (β = −0.381, confidence interval = −0.564 to −0.199, *P* = 4.19 × 10^-5^, FDR = 0.031). SMR analysis, MAGMA Gene-set analysis, and genes mapped on the Functional Mapping and Annotation platform jointly identified HLA-DQA1, HLA-DQB1, IL12RB2, and AIF1. Molecular docking studies forecasted prospective drug-target engagements, reinforcing their therapeutic promise. Our study indicates that an increased proportion of IgD- CD27- %lymphocytes lowers the risk of PBC and explores the potential pathogenic mechanisms of PBC through genetic means, charting a course for the discovery of novel pharmacological targets and delineating new prospects for drug development.

## 1. Introduction

Primary biliary cholangitis (PBC) is a chronic progressive autoimmune cholestatic disease characterized by non-suppurative inflammation of the small intrahepatic bile ducts, with the liver as the primary target organ. This pathological process ultimately results in hepatic fibrosis and cirrhosis.^[1,2]^ PBC is a worldwide disease that predominantly affects middle-aged women aged 40 to 60.^[3]^ Prior case-finding studies have demonstrated a median female-to-male ratio of 10:1.^[4]^ The global incidence of PBC is 1.76 per 100,000, with a total prevalence of 14.60 per 100,000, with the highest rates observed in North America and the Nordic countries. Despite PBC being classified as a rare disease, its incidence has been progressively increasing in recent years.^[5]^

The pathogenesis of PBC is intricate and remains incompletely understood, but it is believed to be influenced by genetic susceptibility, environmental factors, and immune dysregulation.^[6]^ The comprehensive investigation into the immunologic pathogenesis of the disease has been a focal point in recent studies. Current attention is directed towards the amplification of autoimmune responses in various T cell subsets, including Th1, Th17, regulatory T cells (Tregs), follicular regulatory T (Tfr) cells and follicular helper T (Tfh) cells, which exhibit heightened autoimmune reactivity towards bile duct epithelial cells and produce substantial quantities of immune-related factors such as IL-17, IL-6, IFN-γ, and TGF-β, thereby expediting the progression of PBC fibrosis.^[7,8]^ Furthermore, the presence of anti-mitochondrial antibodies and elevated levels of serum immunoglobulin (IgM) in patients with PBC indicates the potential involvement of B cells in the pathogenesis of PBC.^[9]^ Beyond T and B lymphocytes, the immune system comprises cell types such as natural killer (NK) cells, natural killer T (NKT) cells, dendritic cells (DCs), monocytes, and macrophages have also been implicated in the pathogenesis of PBC.^[10]^

PBC exhibits genetic susceptibility, as evidenced by the relative risk of developing PBC in first-degree relatives of patients with familial PBC, which ranges from 9.13 to 10.5, and a concordance rate of 63% in identical twins, marking the highest genetic correlation observed in autoimmune diseases.^[11]^ Since the early 2000s, genome-wide association studies (GWASs) have emerged as the primary comprehensive approach for investigating disease-susceptibility genes associated with polygenic complex traits. Mendelian randomization (MR) serves as an effective strategy to identify causal relationships between exposures (immune cells) and outcomes (PBC), leveraging genetic variants from GWAS summary data as instrumental variables (IVs).^[12]^ Single-nucleotide polymorphisms (SNPs) that exhibit strong correlations with specific exposures, when used as IVs, can mitigate the influence of confounding factors, as they are based on random Mendelian genetic variation.^[13]^ Functional analyses of the identified genes are conducted to achieve a more comprehensive understanding of the mechanisms underlying disease development. GWAS assists in elucidating the genetic architecture of PBC and identifying associated genomic loci and risk genes. Additionally, summary‑data‑based MR (SMR) analysis has been employed to explore new therapeutic targets against the primary pathogenesis of PBC.

## 2. Methods

### 2.1. Study design

**Figure 1.**
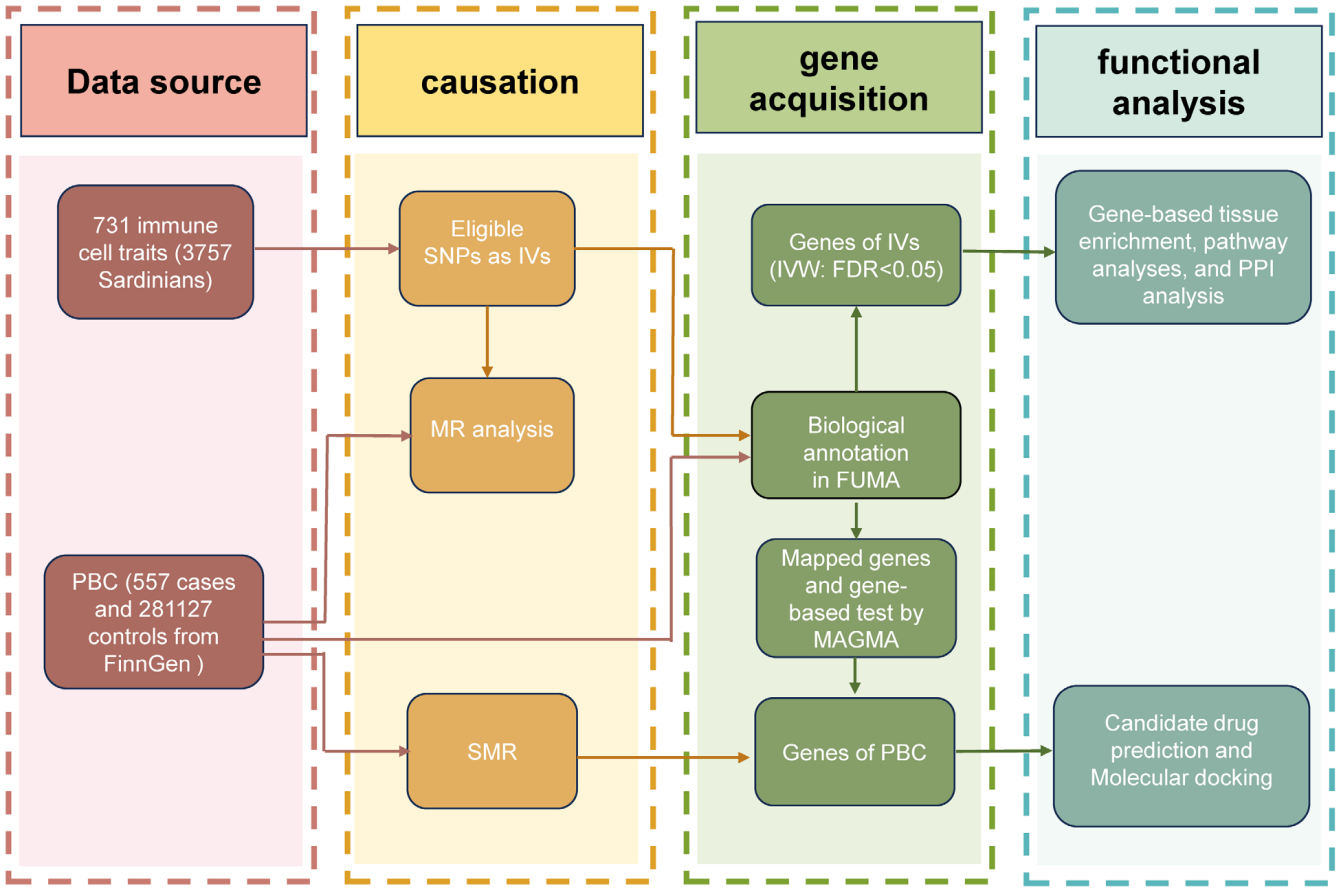
Overview of the study design.

Our analysis explored the causal relationship between 731 immune cell characteristics and PBC utilizing a 2-sample MR framework. In causal inference, MR employs SNPs to represent specific exposure. Therefore, valid IVs must satisfy 3 necessary assumptions: association with the exposure factor, independence from confounding factors, and exclusive contribution to the outcome through the exposure factor without involvement in other pathways.^[14]^ Subsequently, functional annotation of SNPs included in the MR analysis was performed to identify genes of interest, gene-based tissue enrichment, pathway analyses, and protein-protein interaction (PPI) analysis were conducted to anticipate the underlying mechanisms. Our study adheres to the STROBE-MR guidelines and the overview of this study was presented in Figure 1.

### 2.2. Data source

The summary statistics of 731 immune cell traits were obtained from the study by Orrù et al.^[15]^ This cohort involving 3757 Sardinians reported the effects of approximately 22 million variants on 731 immune cell characteristics, illuminating the intricate genetic regulation of immune cells, pinpointing highly specific impacts on the risk of autoimmune diseases at the cellular subtype spectrum. The study detailed the immunophenotype analysis, encompassing absolute cell counts (AC, n = 118), surface antigen levels via median fluorescence intensities (MFI, n = 389), morphological parameters (MP, n = 32), and relative cell counts (RC, n = 192). And classified by cell type as B-cells, cDCs, mature T cells, monocytes, myeloid cells, Tregs, and T-B-NK (T cells, B cells, natural killer cells) across 7 panels (Supplementary Table S1). Data pertinent to PBC from the FinnGen (release 9, available at r9.finngen.fi) were leveraged, identifying PBC cases via the ICD-10 code K74.3, encompassing 557 individuals diagnosed with PBC against a control group of 281,127. Exposure and outcome data were all from European ancestry populations and there was no sample overlap.

### 2.3. Mendelian Randomization analysis

To obtain more IVs, we selected genetic variants associated with each immune cell traits below a less stringent significance threshold of *P* < 1 × 10^-5^, followed by pruning at an r2 threshold of 0.01. The potency of each IV was quantified through the F-statistic, determined by the formula: F = R^2^ (N − 2)/(1–R^2^), where N represents the sample size of the exposed data and R^2^ represented the extent to which the exposure could be explained by the SNP. The formula for R^2^ can be expressed as: R^2^ = 2 × EAF × (1-EAF)×β.^[2]^ We included only IVs with an F-statistic > 10 to ensure that the study results would not be influenced by weak instrumental variable bias. MR analyses were carried out in each immune cell traits to investigate its causal associations with PBC. The Inverse Variance Weighted (IVW) model, our chief MR approach, delivers reliable causal estimates when all 3 assumptions for IVs are satisfied, marking it as the most robust MR method. Supplementary analyses were conducted using the Weighted Median and MR-Egger methods. For the sake of the validity of our findings, we performed sensitivity analysis: Heterogeneity test: Due to the MR analyses of exposures and outcomes from 2 different samples, there may exist population heterogeneity. To ascertain potential assumption deviations through heterogeneity among individual IVs, the Q-test was applied within both IVW and MR-Egger frameworks. A significance threshold of *P* < .05 signals heterogeneity presence.^[16]^ And we use a randomized model. Pleiotropy Test: When the exposure factor is 0, the IVs should still be effective on the outcome, indicating its action through confounders. Utilizing the MR-Egger intercept, horizontal pleiotropy was evaluated. An intercept at 0 on the scatterplot’s Y-axis (*P* > .05) indicates no horizontal pleiotropy.^[17]^ Leave-one-out: By iteratively excluding each incorporated SNP and assessing the magnitude of the effect size changes, we can examine whether the conclusion is significantly impacted by individual SNP.^[12]^ Since our MR analysis performed multiple tests, false discovery rate (FDR) correction strategies were implemented to regulate the ratio of true positives to false positives within a predefined threshold. An association was deemed statistically significant when the causal effect estimate for any immune cell traits showed an FDR of <0.05. The MR analyses were conducted using the TwoSampleMR package, version 0.4.25, operational within the R computational framework, version 4.2.2.

### 2.4. Biological annotation

Employing the Functional Mapping and Annotation (FUMA) platform facilitated the mapping of SNPs to genes and the delineation of linkage disequilibrium (LD)-independent genomic regions, adhering to established protocols.^[18]^ This process involved mapping all genes within a 10 kb radius of each variant. We defined Independent Significant SNPs (IndSigSNPs) as those achieving genome-wide significance (*P* ≤ 5.0 × 10^-8^) and demonstrating mutual independence (r2 < 0.6). From these, Lead SNPs were categorized as IndSigSNPs in LD at r2 < 0.1 within a 500 kb range. By aggregating Lead SNPs within 250 kb proximity, genomic risk loci were established. Clumping was executed utilizing the European 1000 Genomes Project phase 3 as a reference, with the major histocompatibility complex locus consolidated into a singular region due to pronounced LD. Parallelly, a gene-based analysis is being conducted through MAGMA, leveraging GWAS summary statistics.

### 2.5. Gene-based tissue enrichment, pathway analyses, and PPI analysis

In FUMA analysis, we conducted gene-based tissue enrichment based on the protein-coding genes identified from genomic risk loci. To investigate the possible mechanisms by which immune cells may contribute to PBC, we selected mapped genes of interest included in the MR analysis to a Gene Ontology (GO) enrichment analysis. Simultaneously, the Kyoto Encyclopedia of Genes and Genomes (KEGG) database facilitated the exploration of involved gene signaling pathways, while PPI networks were elucidated via STRING version 11.^[19]^

### 2.6. SMR analysis applied to PBC evaluation

The SMR analysis, performed with SMR software version 1.3.1,^[20]^ is designed to explore the potential colocalization between GWAS signals and expression quantitative trait loci (eQTL) data. This methodological approach aims to correlate gene expression levels directly to a phenotype of interest, integrating GWAS summary statistics with eQTL information. For this analysis, data from 3 eQTL sources were incorporated: CAGE blood eQTL dataset (n = 2765),^[21]^ and GTEx v8 datasets for blood (n = 670) and liver (n = 208) eQTLs.^[22]^ Adjustments for multiple comparisons were made using the FDR correction method. Furthermore, a HEIDI test with a *P* value > 0.01 was indicative of the protein-PBC association not being influenced by linkage disequilibrium.

### 2.7. Candidate drug prediction

In our quest to uncover viable drug candidates for BPC, we systematically analyzed genes implicated in BPC across various studies. This comprehensive gene list was cross-referenced against the Drug Signatures Database (DSigDB, https://dsigdb.tanlab.org/DSigDBv1.0/),^[23]^ a robust repository containing 22,527 gene sets and 17,389 unique compounds, mapping to 19,531 genes. DSigDB stands at the forefront of translational research tools, enabling the interrogation of vast gene-drug interaction landscapes to discern compounds with potential therapeutic efficacy against specific genetic profiles. This approach facilitated a targeted search for drug candidates, leveraging the database’s extensive compound collection to forecast therapeutic responses based on the genetic underpinnings of PBC.

### 2.8. Molecular docking

For the molecular docking phase, we initiated our analysis by acquiring the structural data of each candidate small molecule from the PubChem database (https://pubchem.ncbi.nlm.nih.gov/) and the 3-dimensional(3D) protein structures of corresponding receptors from the RCSB Protein Data Bank (PDB, https://www.rcsb.org). Preparatory work on the protein structures was meticulously conducted using PyMOL, a molecular visualization system that affords precise manipulation of biomolecular structures. Utilizing PyMOL, we prepared the protein structures for docking by removing extraneous water molecules and ligands, then adding polar hydrogen atoms to ensure accuracy in our simulations. Subsequent to this preparation, molecular docking simulations were conducted employing AutoDock Vina (version 1.1.2), which facilitates the identification of optimal ligand-receptor binding poses through a sophisticated scoring function that estimates binding affinities, thus indicating the strength and stability of the interaction. This meticulous preparation underpins our molecular docking process, aiming to elucidate the binding affinities and interactions between our drug candidates and target proteins.

## 3. Results

### 3.1. Causal effects of immune cell on the PBC outcomes

We performed MR analysis of 731 immunophenotypes with PBC in which a total of 51 meaningful immune cell traits were identified (Supplementary Table S2), which were detected after FDR adjustment to be protective against the development of PBC only in the B-cell panels IgD- CD27- %lymphocyte (β = −0.381, CI = −0.564 to −0.199, *P* = 4.19 × 10^-5^, PDR = 0.031). The 22 SNPs we identified were strongly associated with IgD-CD27- %lymphocyte, with F-statistic all > 10 indicating strong statistical strength of the selected IVs, and strongly associated with the immune cell traits (Supplementary Table S3). Sensitivity analysis showed that no heterogeneity was observed (P_Q_ > 0.05). The P-values of MR-Egger intercept tests were > 0.05 did not demonstrate directional pleiotropy. Additionally, the leave-one-out method also did not identify a single SNP that affected the results. (Supplementary Figure S1)

### 3.2. Genomic loci and mapped genes identification

**Figure 2.**
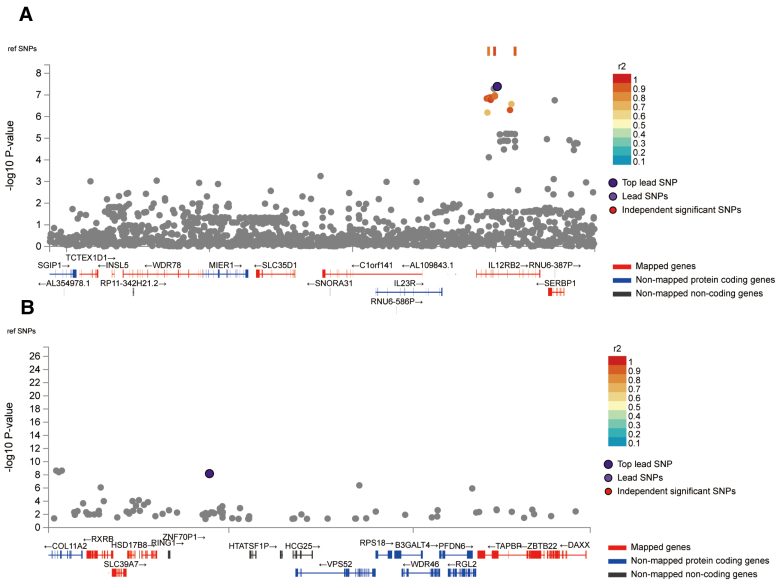
(A) The 1p31.3 genomic locus of the PBC GWAS data. (B) The 6p21.32 genomic locus of the PBC GWAS data.

The IVs (22 SNPs in total) for the immune cell traits and PBC with potential causal relationships in 2-sample MR identified 64 genes (Supplementary Table S4). The positional mapping, eQTL-based mapping, and chromatin interaction-based mapping implicated. The GWAS data of PBC identified 2 genomic loci 1p31.3 and 6p21.32 (Fig. 2), which include 17 protein-coding genes (Table1). Gene-based test conducted by MAGMA as illustrated in Supplementary Figure S2.

### 3.3. Tissue enrichment, pathway and PPI analyses

**Figure 3.**
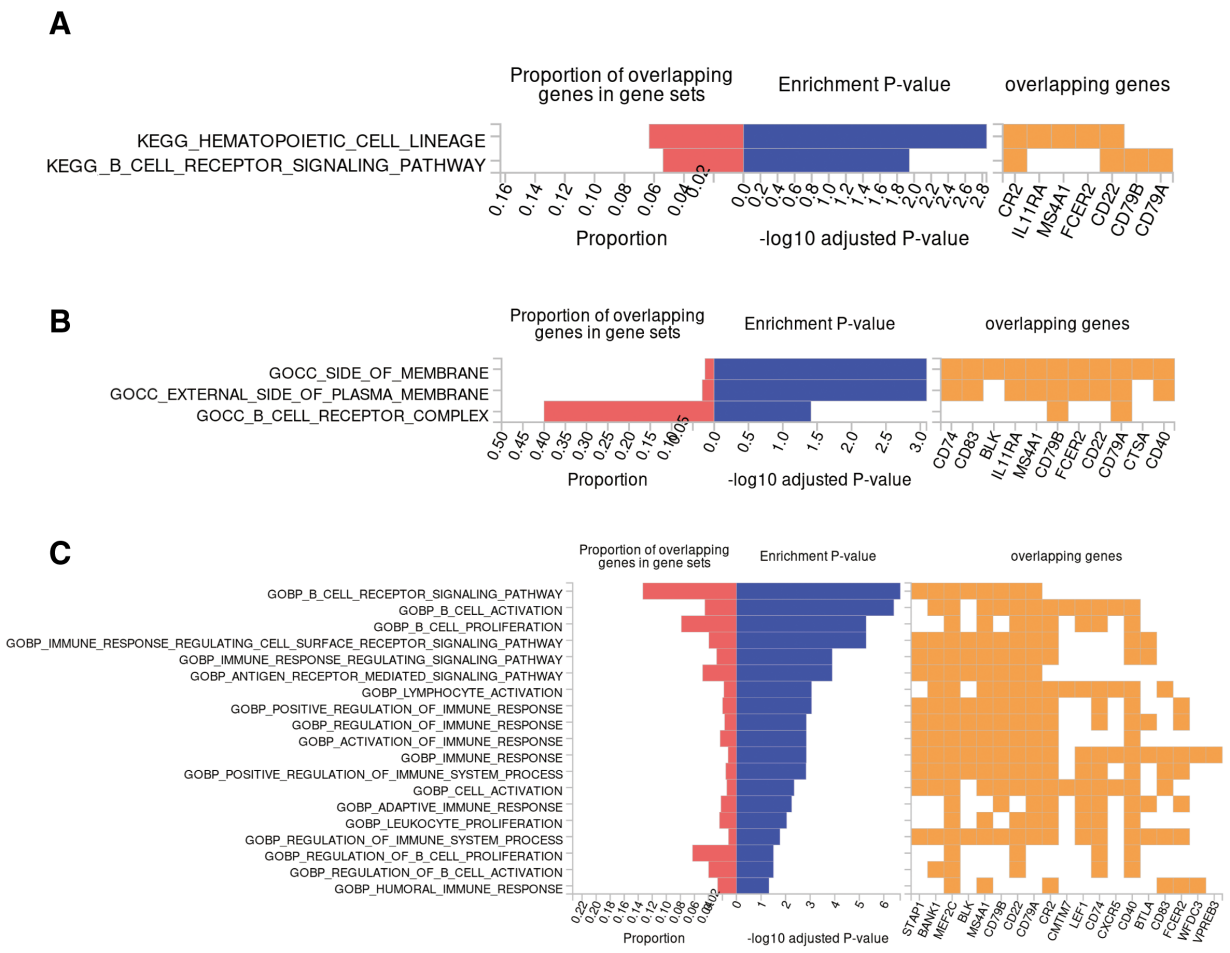
Pathway analyses of immune cell-related genes causally associated with PBC. (A) KEGG analysis of the 64 genes. (B) GO cellular components analysis of the 64 genes. (C) GO biological processes analysis of the 64 genes analysis.

**Figure 4.**
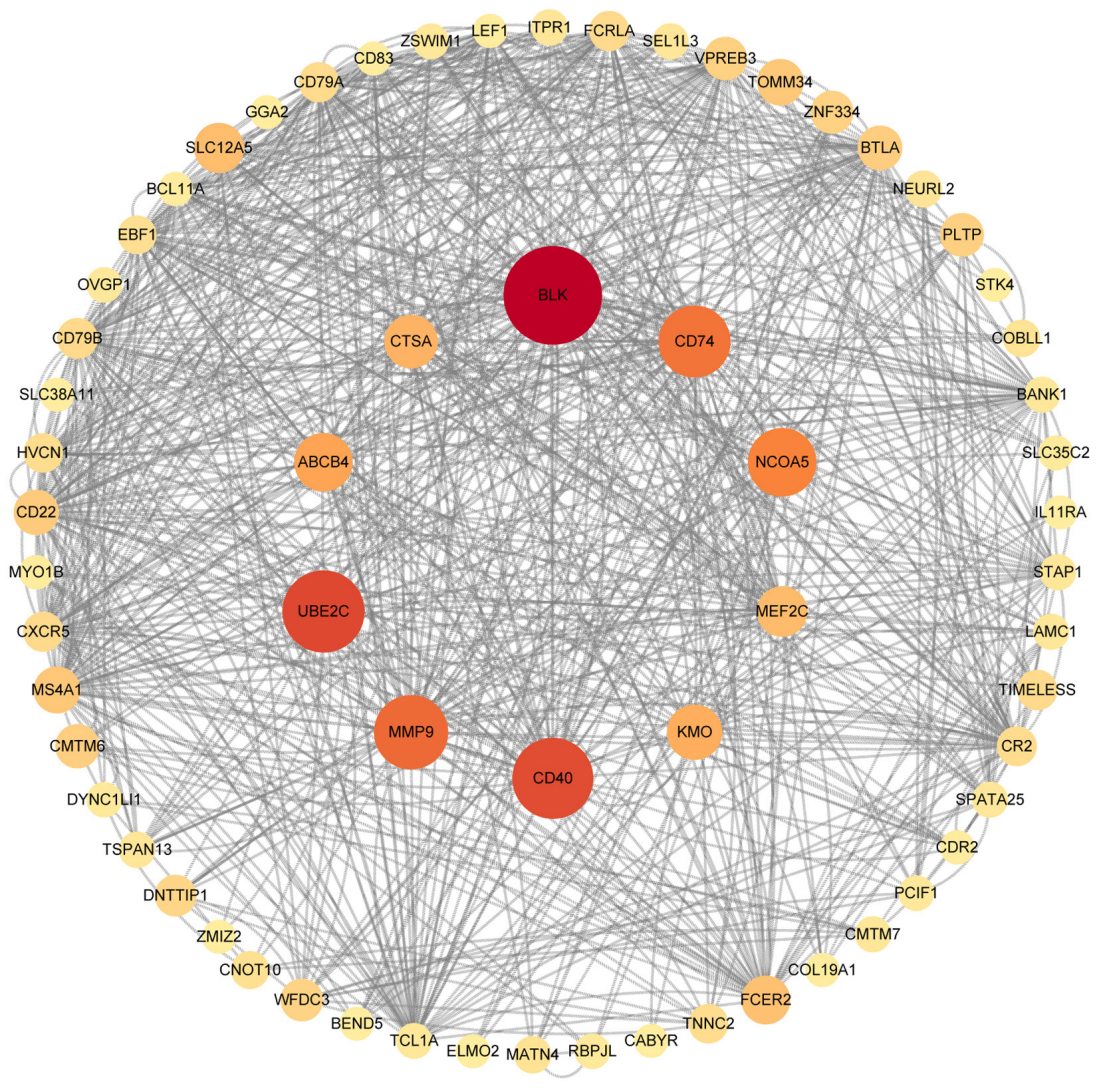
PPI network built with STRING.

In the tissue enrichment analysis, we excluded genes located via Trans-eQTL(eQTLGen_trans_eQTLs), which refers to eQTLs regulating genes located far away, over 5Mb apart, and sometimes not even on the same chromosome. For example, DNA segments of co-transcription factors regulating this gene. Ultimately, 25 genes located on chr3p22 and chr20q13 were found to be downregulated in liver tissue (Supplementary Figure S3), utilizing GTEx v8 with 30 general tissue types as the reference panel, which consistent with the results of our MR analysis. Pathway analyses using KEGG revealed that the set of 64 genes primarily participates in the hematopoietic cell lineage and B cell receptor signaling pathways. Similarly, GO-based analysis corroborated these genes’ engagement in biological processes pertinent to B-cell proliferation, activation, and immune response regulation, as well as in the cellular components of plasma membrane, cell receptor and immunoglobulin complex (Fig. 3). The PPI analysis utilizing the STRING database revealed a tightly interconnected network formed by the 64 proteins and we obtained 10 hub genes ranked by Betweenness method, which were analyzed and visualized via Cytoscape (V3.8.2). Consistent with the previous enrichment analysis, network functional analysis similarly highlighted a strong relevance to immune function, aligning with the autoimmune nature of PBC (Fig. 4).

### 3.4. SMR analyses of PBC

To identify genes of functional significance, SMR analysis on PBC data was conducted utilizing 3 distinct eQTL datasets, which involved 10 specific genes (Supplementary Table S5 and Figure S4). Among them, HLA-DQB1, HLA-DQB1-AS1, HLA-DQA1 and HLA-DQB2 were implicated by GTEx v8 liver and blood eQTL and IL12RB2 by GTEx v8 blood was consistent with the protein-coding genes identified by the risk loci in FUMA. There is compelling evidence of a strong association between the expression of human leukocyte antigens (HLA) and IL12RB2 and the occurrence of PBC.

### 3.5. Candidate drug prediction and molecular docking

**Figure 5.**
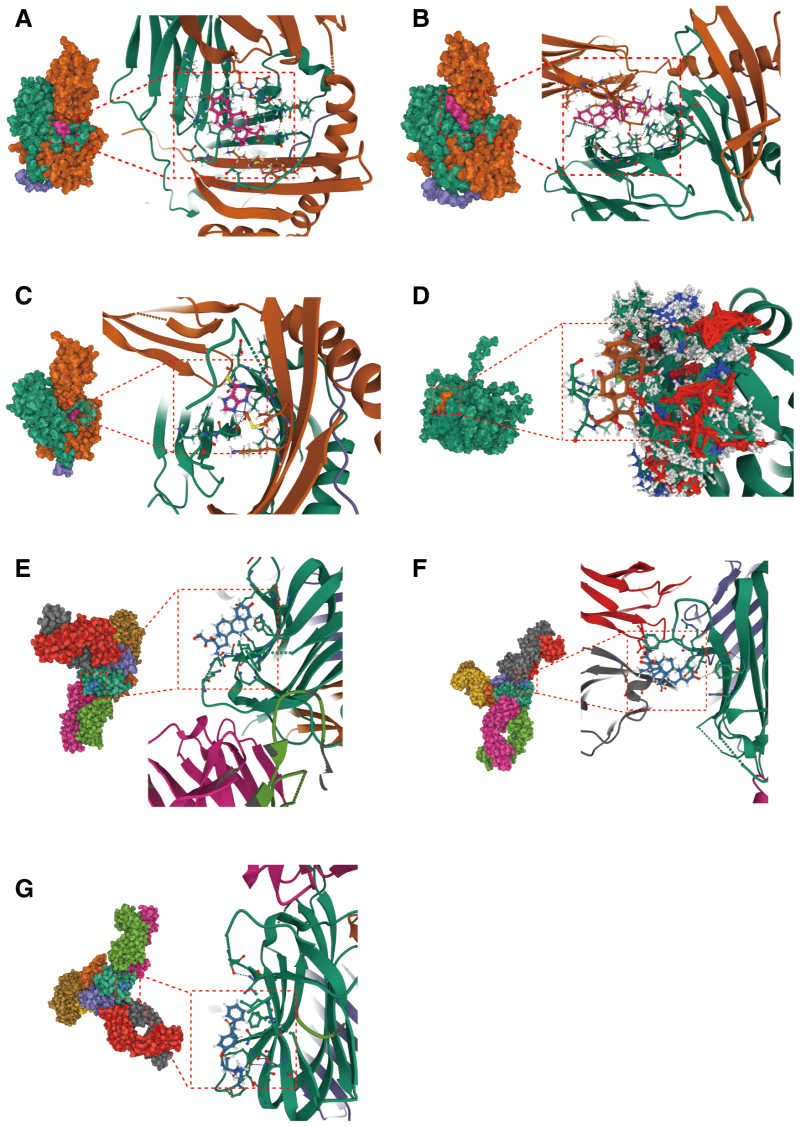
Molecular docking results of available proteins small molecules. (A) HLA-DQA1~ Dexamethasone (B) HLA-DQA1~ Testosterone enanthate (C) HLA-DQA1~ 6-Mercaptopurine (D) AIF1~ Dexamethasone (E) CD40~ Dexamethasone (F) CD40 ~ Betamemethasone valerate (G) CD40~ Tesmilifene.

In this investigation, predictions regarding efficacious pharmacological interventions were derived using the DSigDB database. The leading 10 chemical compounds, as determined by adjusted *P*-values, are delineated in Supplementary Table S6. Our findings reveal that dexamethasone (CTD 00005779) interacts with a broad spectrum of genes, most notably targeting the HLA-DQA1 and HLA-DQB1 loci. Due to the absence of the human IL12RB2 protein’s 3D structure in the PDB database, our analysis was confined to the binding sites of proteins encoded by 4 specific genes and the interaction with several small molecules of potential target drugs (Fig. 5). Notably, AIF1 and dexamethasone demonstrated the lowest binding energy (−67.282 kcal/mol) (Supplementary Table S7), suggesting a highly stable interaction.

## 4. Discussion

Human immune cells play a pivotal role in the immune system, safeguarding physiological equilibrium and promoting self-tolerance to avert and combat illnesses. These cells, derived from pluripotent hematopoietic stem cells within the bone marrow, diversify into lymphocytes (notably T cells, B cells, and NK cells) and myeloid lineage cells (such as neutrophils, eosinophils, basophils, dendritic cells, mast cells, and monocytes/macrophages). This differentiation process also gives rise to erythrocytes and megakaryocytes, the latter of which are responsible for platelet production. Immune cells are governed by intricate genetic regulatory frameworks, with CD4 T cells, CD8 T cells, and B cells demonstrating the most pronounced genetic predispositions.^[24]^ These cells exhibit highly selective effects on autoimmune disease risk at the cell-subtype leve.^[15]^ PBC, an autoimmune liver condition, involves immune cell infiltration around small bile ducts, contributing to ductal damage and playing varied roles in disease progression.

We conducted this study to evaluate the causal effects of 731 immune cell traits on PBC risk using MR analysis, aiming to identify immune cell gene expressions and functionalities associated with PBC, while also exploring the genetic mechanisms of PBC. Our analysis indicated a notable decrease in PBC risk associated with higher levels of IgD-CD27- %lymphocytes in the B cell panel. B cells are generally believed that contribute to the immunopathogenesis and maintenance of PBC.^[25]^ CD27 absent in naive B cells, serves as a pivotal marker for identifying human memory B cells. As a constituent of the tumor necrosis factor (TNF) receptor superfamily, CD27 engages in interactions with CD70 on activated helper T cells, facilitating the transition of memory B cells into plasma cells.^[26]^ Moreover, the CD27:CD70 axis plays a crucial role in the activation of CD4 + T lymphocytes. Notably, in PBC patients, there is a marked overactivation of CD4 + T cells within the peripheral blood.^[27]^ During the early stages of PBC, a targeted accumulation of autoreactive CD4 + T lymphocytes occurs within the liver tissue, with these cells uniquely responsive to the E2 subunits of the pyruvate dehydrogenase complex (PDC-E2). This accumulation heralds a predominance of Th1 cell responses, characterized by the secretion of cytokines such as IL-2 and IFN-γ.^[28,29]^ These responses initiate a pro-inflammatory cytokine-driven feedback loop, culminating in sustained autoimmune inflammation within the PBC-affected liver. As PBC progresses, the inflammatory mechanism evolves from Th1 to Th17 cell differentiation, with a consequent increase in IL-17 secretion, a finding consistent in peripheral blood.^[30]^ Employing multiplex immunofluorescence (IF) on frozen liver biopsies, Li et al reported a pronounced accumulation of CD27 memory B cells in the portal areas of PBC patient livers, markedly exceeding that in controls.^[31]^

Biological annotation analyses of SNPs with potential causal relationships identified through 2-sample MR revealed 64 protein-coding genes. Pathway enrichment analysis revealed that the genes are mainly related to immune responses, especially in the roles of B cell proliferation and activation. The increase of IgD-CD27- %lymphocytes appears to be a protective factor for PBC, lowering disease risk by downregulating genes involved in B cell proliferation and activation in the liver. Among the 10 hub genes identified, CD40 was highlighted, a gene previously extensively reported in patients with PBC. CD40, a member of the Tumor Necrosis Factor Receptor Superfamily, plays a crucial role in the immune system as a receptor on antigen-presenting cells, essential for mediating a wide range of immune and inflammatory responses.^[32]^ Interferon-gamma (IFN-γ) and CD40 ligand (CD40L) are identified as the most significant upstream regulators in PBC.^[33]^ In PBC, CD40 mediates bile duct epithelial apoptosis through the Fas/FasL pathway, while members of the Tumor Necrosis Factor (TNF) receptor family amplify the apoptotic process.^[34,35]^ Additionally, CD40 and CD27 play a role in the genetic regulation of B cells within autoimmunity contexts. The rs1883832[T] variant in CD40’s 5′ untranslated region enhances CD27 trans expression on memory B-cell subsets and reduces a particular B-cell subset lacking CD27 expression. This genetic variation is linked to a heightened risk for diseases such as multiple sclerosis, systemic lupus erythematosus, Crohn disease, inflammatory bowel disease and chronic hepatitis infection. Conversely, it is associated with a lower risk of developing Kawasaki disease and rheumatoid arthritis (RA).^[15,36–39]^ In an animal study, administering anti-CD40L antibody (anti-CD40L) to mice delayed the progression of autoimmune cholangitis. Although the effect diminished over time, further research into the mechanisms involving the CD40 target remains crucial for the treatment of PBC.^[40]^

The SMR analysis of PBC identified 17 risk genes for the disease, including several human leukocyte antigen (HLA) genes. The HLA genes, located in the densest genomic region at chromosomal position 6p21,^[41]^ represent the most genetically diverse loci in the human genome. HLA molecules play a critical role in the immune response under physiological conditions and in inducing autoimmune reactions in disease states. A considerable body of research has reported on the involvement of HLA molecules in the pathogenesis of PBC.^[42]^ The MAGMA gene-based analysis lends functional insights into several genes identified with genome-wide significance in PBC studies, including HLA-DQA1, HLA-DQB1, and AIF1. Additionally, SMR analysis and FUMA gene mapping identified a shared gene, IL12RB2. During the apoptosis of biliary epithelial cells, PDC-E2 is internalized through endocytosis, precipitating antigen-presenting cells’ maturation alongside the secretion of cytokine IL-12.^[43]^ IL-2 activates a series of signaling molecules, such as Nuclear Factor-kB (NF-kB) and STAT4, through its receptor subunit IL12RB2, promoting the production of pro-inflammatory Th1 cytokines, including TNF-α and IFN-γ, and enhancing the cytotoxic responses of NK, NKT, and CD8 + T cells.^[44]^ A GWAS by Hirschfield et al demonstrated a significant association between PBC and class II HLA genes, with the strongest association observed at the HLA-DQB1 locus, and common genetic variants at the IL12A and IL12RB2. This indicates that the IL-12 immunoregulatory signaling axis is relevant to the pathophysiology of PBC, findings that are highly consistent with our research.^[45]^

Ursodeoxycholic acid (UDCA) remains the first-line medication for treating PBC, offering choleretic effects that promote bile secretion, inhibit the cytotoxic actions of hydrophobic bile acids and their induced apoptosis, providing cellular protection, anti-inflammatory, and immunomodulatory benefits. Thus, it protects biliary and liver cells, improving biochemical markers, alleviating pathological changes, and slowing disease progression. However, patients with early histological changes may benefit more, with around 40% of patients showing incomplete or no response to UDCA.^[46]^ Our drug predictions highlight the therapeutic potential of these genes, with molecular docking showing high binding activity, indicating these genes as promising drug targets. Notably, dexamethasone demonstrated good affinity across multiple gene sites. Studies have shown that combining UDCA with dexamethasone upregulates human AE2 alternate overlapping promoter sequences from which AE2b1 and AE2b2 are expressed, stimulating hepatocyte-mediated AE2 excretion of bicarbonate, thereby promoting the excretion of bicarbonate-rich bile.^[47]^ Furthermore, molecular docking results reveal considerable binding affinity of certain compounds, highlighting these genes’ viability as therapeutic targets.

Despite the significant findings of our study, it is crucial to acknowledge several limitations: Genetic susceptibility to PBC varies among different populations, and our research analyzed samples representing only the European population. To strengthen the validity and applicability of our conclusions, further analyses incorporating GWAS results from other populations with PBC are needed. Mendelian randomization is applicable only to risk factors with suitable genetic variants. Given that PBC is also influenced by environmental factors, if genetic variants explain only a small proportion of the variance, this may result in lower statistical power or even false-negative outcomes for MR analysis. It is necessary to use multiple genetic variants related to risk factors as instrumental variables to increase the proportion of variance explained and thus improve statistical power. Molecular docking elucidates potential ligand-receptor engagements, yet its practicality and efficacy are confirmed via additional experimental exploration and clinical trials.

## 5. Conclusions

In summary, our MR results suggest that an increased proportion of IgD-CD27- %lymphocytes leads to a decreased risk of PBC, potentially through the downregulation of genes related to B cell proliferation and activation. Additionally, our research furnishes functional validation for several established risk genes associated with pathogenesis, reveals new risk genes for PBC, and highlights the genetic mechanisms contributing to the disease’s onset.

## Acknowledgments

The authors express their gratitude towards several key contributors and supporters of their research. They thank the FinnGen consortium and the team behind a significant GWAS led by Orrù et al for their valuable data and insights. The authors also show appreciation for the FUMA platform developers, including Kyoko Watanabe, for their indispensable tools and resources.

## Author contributions

**Conceptualization:** Yukai Lin, Jianfeng Mei.

**Data curation:** Hao Chen.

**Formal analysis:** Yukai Lin, Haifei Tang.

**Investigation:** Hao Chen.

**Methodology:** Yukai Lin, Haifei Tang.

**Project administration:** Yanming Hua, Jianfeng Mei.

**Software:** Xiaolong Xu.

**Validation:** Xiaolong Xu.

**Writing – original draft:** Yukai Lin.

**Writing – review & editing:** Xiaolong Xu, Hao Chen, Yanming Hua, Jianfeng Mei.















**Figure:**
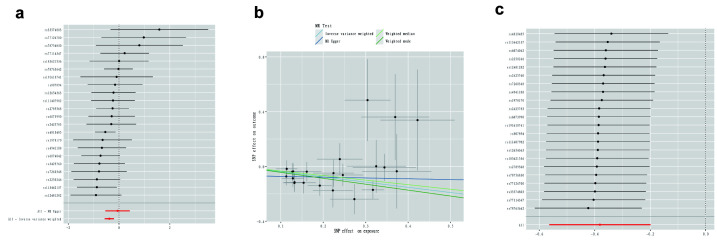


**Figure:**
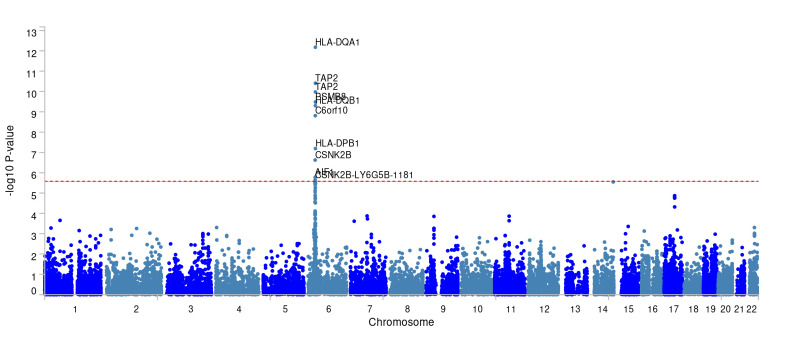


**Figure:**
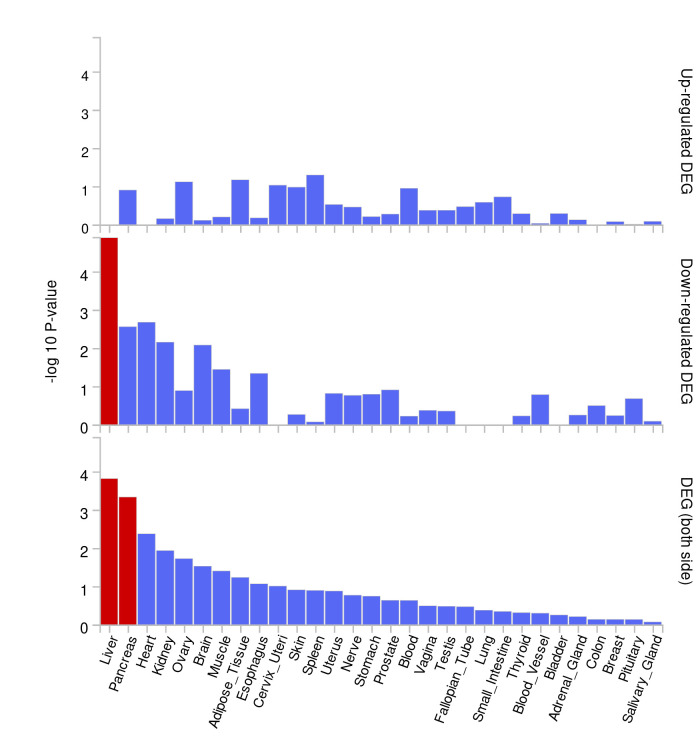


**Figure:**